# The involvement of osmolarity in the safety of contrast media

**DOI:** 10.1186/s12948-018-0097-4

**Published:** 2018-09-03

**Authors:** Stefania Isola, Fabiana Furci, Sebastiano Gangemi

**Affiliations:** School and Operative Unit of Allergy and Clinical Immunology, Department of Clinical and Experimental Medicine, University of Messina, Messina, Italy

**Keywords:** Contrast media, Osmolarity, Hypersensitivity, Adverse drug reaction

## Abstract

**Background:**

New non-ionic contrast agents, classified into low osmolar agents and iso-osmolar agents, present different biochemical characteristics that may influence the allergic reactions they cause. The aim of our study was to evaluate how osmolarity may affect safety in the use of contrast agents.

**Case presentation:**

Six patients with a positive history for reaction to contrast agent were included in this study. Only one patient prick and intradermal skin test was positive. However, in 5 cases, patients presented an immediate reaction after administration of contrast agent that was not IgE mediated.

**Conclusions:**

In this study, we focused on iodixanol, an iso-osmolar contrast agent, finding good safety of this product in patients with previous hypersensitivity reactions to contrast agent.

## Background

The use of intravenous contrast media (CM) allows the opacification of vessels and tissues while performing a radiological examination, with the purpose of providing information to evaluate a patient’s clinical problem.

Iodinated CM are concentrated solutions of tri-iodinated benzene derivatives and were introduced into clinical practice in the 1950s. These medications are used for X-ray examinations and for computed tomography (CT) [1]. Iodinated CM are classified into ionic and non-ionic types. New non-ionic CM are classified into monomeric low osmolar agents, such as iohexol, iopamidol, iopromide, ioversol, iomeprol, iobitridol and ioxilan, and dimeric iso-osmolar CM, such as iodixanol.

Cross-reactivity between iodine-based contrast agents was reported more frequently in subjects with delayed reactions than in subjects with immediate reactions [2–5] and seemed to depend, at least in part, on the chemical structure of the compounds [6]. A recent study [5] proposed a classification of contrast agents into subgroups based on the most frequent cross-reactivity patterns:

Subgroup A: ioxitalamate, iopamidol, iodixanol, iomeprol, ioversol, iohexol.
Subgroup B: iobitridol, ioxaglate.
Subgroup C: amidotrizoate.

CM are also used for magnetic resonance imaging (MRI); the most used are gadolinium chelates or complexes [7, 8]. These molecules are classified according to net charge, ionic or non-ionic, and structure, linear or macrocyclic.

Adverse reactions after CM administration have been divided into allergic and non-allergic hypersensitivity, toxic reactions (e.g. nephrotoxicity, neurotoxicity), and reactions unrelated to CM application [9]. The two severity scales of Ring and Messmer or Brown can be used to classify reactions [10].

Hypersensitivity reactions (HSR) are classified in immediate, if they appear within the first hour after administration, and in non-immediate, if they appear from more than 1 h to several days after administration [11].

While these reactions are generally mild in nature, 1% to 3% of patients exposed ionic contrast agents and 0.05% of patients exposed to non-ionic contrast agents have severe reactions and most are immediate [12]. An immunoglobulin E (IgE)-mediated allergic mechanism for hypersensitivity to CM can be demonstrated only in a minority of cases. Other mechanisms are involved in the pathophysiology of these reactions; in particular, for immediate cases it may be a direct membrane effect, possibly related to the osmolarity of the CM solution, with histamine release from basophils and mast cells. There are reports of high levels of tryptase in connection with severe or fatal reaction [13, 14]. Non-immediate HSRs induced by CM are instead T cell mediated [15].

The most common symptoms of immediate reactions are pruritus, urticaria, angioedema and flush. Other symptoms include dyspnoea (bronchospasm, laryngeal oedema), nausea, diarrhoea, rhinitis, hypotension, tachycardia, cardiovascular shock, cardiac arrest, respiratory arrest [14–18]. The most common symptoms of non-immediate HSRs are macular or maculopapular eruptions; other less frequent manifestations of non-immediate HSRs may be Steven-Johnson syndrome, toxic epidermal necrolysis, acute generalized pustolosis and vasculitis [15, 19].

Clinical manifestations, such as heat, facial flushing and nausea, that generally occur after administration of CM, not associated with other symptoms and which do not require medical therapy, usually resolve spontaneously and are not suggestive of allergic reactions [20].

The most frequent risk factor for an immediate HSR is a previous immediate reaction to CM. Other risk factors for more severe immediate reactions include severe allergy, bronchial asthma, concomitant medications (e.g. ACE-inhibitors, B blockers, proton pump inhibitors), cardiac disease.

Reported predisposing risk factors for non-immediate HSRs include previous CM-induced adverse reaction, serum creatinine level > 2.0 mg/dl, interleukin-2 treatment, a history of drug and contrast allergy.

Other important risk factors are: viral infections at time of CM exposure, autoimmune disease and mastocytosis [21–26].

The evaluation of patients with reactions to CM can be initiated during the acute phase with serum tryptase (at the onset of the reaction and 2 and 24 h later) [27, 28]. A twofold increase in tryptase is indicative of anaphylaxis [29].

The basophil activation test, used to detect basophil activation markers (CD45, CD18, CD63) and the lymphocyte activation test, based on the ability of T-cell to proliferate upon contact with CM in sensitized patients, are vitro methods whose sensitivity and specificity has not yet been established [30, 31].

In patients with a positive history of reaction to CM, the drug provocation test (DPT), considered the gold standard for the diagnosis of adverse drug reactions (ADRs), plays a key role in confirming or excluding the diagnosis when there is no other available evidence and can be used to find an alternative CM. The DPT is performed by increasing doses of the CM (5,15,30 and 50 cc) every 30–45 min for immediate HSRs, and every 60 min for non-immediate HSRs [2].

The current management of patients with CM hypersensitivity who need CM studies requires the use of non-ionic and iso-osmolar CM [32]. This approach was confirmed in our study, finding good tolerability in the use of an iso-osmolar contrast agent (iodixanol-Visipaque) available at our university hospital.

In light of evidence of reactions to CM, which frequently occurred on first exposure, the role of osmolarity may be considered one of the most important factors to study in the choice of which alternative CM to use.

## Case presentations

This study is based on analysis of data from patients with previous HSRs to CM, admitted to the Allergy and Clinical Immunology Unit of the University of Messina. The allergological investigation was performed within 6 months of the adverse reaction to CM. After an accurate medical history, according to the diagnostic procedure of the Drug Allergy Interest Group/European Network of Drug Allergy (DAIG)/(ENDA), skin tests were performed on patients who came to our observation with reaction to CM [3]. Clinical data were recorded using an adaptation of the ENDA drug allergy questionnaire [33].

We observed 6 patients, three with a well-documented history of iodixanol, iomeprol and iopromide HSR and three patients who showed a reaction to unspecified CM. The patients came to our observation due to an adverse reaction to a CM and the need to perform a radiological examination with contrast agent; it was not possible to carry out an alternative radiological examination and hence, an alternative CM needed to be identified. None of the patients who came to our evaluation had allergic-relevant comorbidities, i.e. active urticaria, symptomatic bronchial asthma, recurrent angioedema, mastocytosis, idiopathic anaphylaxis.

We performed skin prick tests (SPT) and intradermal tests (IDT) on the volar forearm and we read them after 20 min and on days 2 and 3. We have reported as positive a SPT if there was a wheal of ≥ 3 mm in diameter after 20 min (immediate reading) or if an erythematous induration occurred at the skin test site on days 2 or 3 (delayed reading). We injected IDT solution (0.03–0.05 ml) into the skin to produce a bleb of 4–5 mm in diameter. The IDT was interpreted as positive if the size of the initial wheal had increased by at least 3 mm in diameter and was surrounded by erythema after 20 min (immediate) or if an erythematous induration at the skin test site was present in the delayed readings. We used histamine (0.01%) and saline (0.9%) as positive and negative controls, respectively. The patients, under hospital surveillance, completed the test with intravenously administration during the next radiological examination starting with a graded dose of the CM taking as a model a study by Soffer et al. [32]. Patients were given 1% of the total expected dose 1 h before radiological procedure and were monitored for 30 min. They then received 10% of the total expected dose, with monitoring for another 30 min. They received the final dose during the radiological examination. This careful monitoring proved to be of considerable importance since previous studies revealed that skin tests do not always predict a CM hypersensitivity reaction [3].

### Case 1

A female patient, 75, was sent to our unit for observation from the general gastrointestinal surgery unit. The patient had a positive history of antibiotic allergy, and a CM (iodixanol) reaction (skin erythema) during coronary angiography, 2 months previous. The cutaneous manifestation occurred after 20 min from administration of the contrast agent, with maintenance of vital parameters. Therefore, the patient was treated with chlorpheniramine 10 mg intramuscular vial plus hydrocortisone 200 mg, intravenously. The cutaneous manifestation disappeared 6 h after therapy.

The clinical history of this patient was silent for inhalants, food allergy and latex allergy. Given that the patient had now to undergo a CT angiography, a challenge test for iodixanol was therefore suggested.

SPTs and IDTs were performed with iodixanol according to the following scheme of administration: SPT 1:10 diluted, SPT undiluted, IDT 1:1000 diluted, IDT 1:100 diluted, IDT 1:10 diluted. The test result was negative. The patient also subsequently tolerated the radiological examination performed using the CM tested, well.

### Case 2

A male patient, 65, suffering from arteriovenous malformation, was sent from the neurosurgery unit as a predetermined adverse reaction (erythematous skin reaction) to an unknown CM had been reported, with normal vital parameters, 3 months previously. The erythematous skin reaction occurred 15 min after administration of the CM: the reaction was resolved 2 h after cortisone and antihistamine therapy, as practiced in case 1.

The patient had a negative history for latex allergy, respiratory allergies, and food allergy.

SPTs and IDTs were therefore performed for iodixanol as in case 1. The test result was negative. The patient also subsequently tolerated the radiological examination performed using the CM tested, well.

### Case 3

A male patient was sent for observation from the hepatology unit with a history of erythema and itching which had appeared immediately, 10 min after administration of an iodinated unspecified CM, 6 weeks previously. The patient did not present impairment of vital parameters. Resolution of skin signs and pruritic symptoms occurred 2 h after cortisone and antihistamine therapy, as practiced in case 1.

He had a negative history for latex allergy, respiratory allergies, and food allergy.

As the patient had now to undergo an abdominal CT with CM, SPTs and IDTs were performed for iodixanol, as in case 1. The test result was negative. Subsequently, the CM used was well tolerated during the execution of the radiological examination.

### Case 4

A male patient, 57, was sent for observation from the neuroendocrinology unit with a reported adverse reaction to an unspecified CM, with appearance of urticaria 20 min after administration of the contrast agent, without compromising vital parameters. The cutaneous manifestation disappeared 5 h after cortisone and antihistamine therapy as practiced in case 1.

The patient had a positive history for allergic rhinitis, and a negative history for food allergy and latex allergy.

SPTs and IDTs were therefore performed for iodixanol as in case 1. The test result was negative. The CM used was well tolerated during the execution of the radiological examination.

### Case 5

A female patient, 67, was referred to us for an allergological evaluation. The patient was undergoing chemotherapy with bevacizumab for ovarian carcinoma, and it was reported that she had presented two episodes of adverse reaction to iomeprol used for the execution of CT examinations.

In both episodes, the patient presented skin erythema, pruritus, facial edema with accentuation of symptomatology and cutaneous manifestations during the second episode, before which she had been pre-medicated with Prednisone 50 mg by mouth at 13 h, 7 h and 1 h before CM injection, plus chlorpheniramine 10 mg intramuscular 1 h before CM [34, 35]. These manifestations, which did not compromise vital parameters, appeared within 10 min of administration of the contrast agent and regressed within 12 h from the cortisone and antihistamine therapy practiced as in case 1; in addition, methylprednisolone 40 mg was administered intravenously.

The patient had a negative history for latex allergy, respiratory allergies, and food allergy.

SPTs and IDTs for iomeprol were performed as in case 1, and the test result was positive. Subsequently, we chose to perform SPTs and IDTs for iodixanol, with negative result. The CM tested was administered and well tolerated during the subsequent radiological examination.

### Case 6

A 67-year-old female patient came to our observation with a history of adverse contrast reaction: 10 years previous, after administration of unspecified CM for abdominal CT, the patient had presented skin rash and swelling of the limb, the injection site of the CM, with maintenance of vital parameters.

A month previous, during angio-CT, 15 min after injection of the CM, iopromide, despite having been premedicated as in case 5, the patient presented erythema and mild edema of the limb in the injection site of the CM and dry cough which lasted for some days. Immediately upon the appearance of the adverse reaction the patient was treated with chlorpheniramine 10 mg intramuscular therapy. In addition to the aforementioned therapy, salbutamol was administered by aerosol. Blood pressure, oxygen saturation and thoracic objectivity were normal.

The patient had a negative history for latex allergy, respiratory allergies, and food allergy. Given the need to repeat angio-CT due to the presence of cerebral aneurysms, SPTs and IDTs were therefore performed with iopromide according to the following scheme of administration: SPT 1:10 diluted, SPT undiluted, IDT 1:1000 diluted, IDT 1:100 diluted, IDT 1:10 diluted. The test results were negative. Subsequently, the CM used was well tolerated during the execution of the radiological examination.

## Discussion and conclusions

In our study, skin tests have proved useful for the confirmation of CM allergy and for identifying a safe alternative product for CM re-exposure. A positive skin test result was found only in one patient, case 5. Subsequently, in this patient iodixanol presented negative result. Cross-reactivity between iomeprol and iodixanol, belonging to the same subgroup but with different osmolarity, was not found. Five patients presented negative skin test results for iodixanol, one patient for iopromide (case 6). All patients, subsequently, tolerated CM tested during radiological examination.

The decreased movement of extracellular fluid osmotically, as well as efflux of the CM may explain the higher tolerability of an iso-osmolar agent, such as iodixanol (Visipaque) [35]. Indeed, Sutton et al. [36] reported that iodixanol is well tolerated in the early phase of injection, constituting a safer CM in relation to immediate HSR. In addition, Gomi et al. [37] reported immediate HSR in 2.7% of patients with iodixanol in comparison to 3.5% with iopromide. Therefore, in agreement with other authors according to our findings, iodixanol presents better compliance and safety for patients with a positive history for immediate HSR [35].

As noted in case 5, there is no certainty regarding the effectiveness of premedication therapy in preventing a possible adverse reaction [34, 38].

This result leads us to hypothesize how osmolarity of the contrast agent is one of the main factors that affects the release of cell mediators and, therefore, the appearance of an adverse reaction. Thus, the osmolarity of a contrast agent may be considered an important factor to be taken into account in those patients who present an immediate reaction to CM and with negative SPTs and IDTs for that particular CM.

Indeed, the importance of drug osmolarity can also be understood from the general medical indications regarding treatment of anaphylaxis, according to which, rapid intravenous administration of isotonic saline is recommended, avoiding, instead, plasma expanders due to the risk of mast cell degranulation [39]. Fluid and oxygen are the most ubiquitous therapeutic interventions in a patient in critical health conditions. The therapeutic administration of fluids aims to expand intravascular, interstitial and intracellular compartments and, in literature, anaphylactoid reactions have been reported with all classes of colloids. These anaphylactoid reactions have an incidence of 0.07%–0.15%, but often such reactions are not reported and therefore misunderstood [40, 41]. Since crystalloid and colloid fluids present different adverse effects, including nephrotoxicity, anticoagulation, acid base disturbance and anaphylactoid reactions, it is important to choose the type of fluid to be administered based on the characteristics of the different types of fluids, their potential effects following administration and the physiologic needs of patients [42].

Thus, with this paper, we would like to further reflect on how a complete evaluation of an individual’s allergic susceptibility and of the biochemical and osmolar characteristics of CM may be of help in choosing the correct CM hypersensitivity management strategy.

More studies are needed to investigate the underlying mechanism of osmolarity in patients with immediate HSR and negative skin tests result.

## Abbreviations

CM: contrast media
CT: computed tomography
MRI: magnetic resonance imaging
HSR: hypersensitivity reaction
DPT: drug provocation test
ADR: adverse drug reaction
DAIG/ENDA: Drug Allergy Interest Group/European Network of Drug Allergy
SPT: skin prick test
IDT: intradermal test
